# Innate Immunity and CKD: Is There a Significant Association?

**DOI:** 10.3390/cells12232714

**Published:** 2023-11-27

**Authors:** Moran Plonsky-Toder, Daniella Magen, Shirley Pollack

**Affiliations:** 1 Pediatric Nephrology Institution, Rambam Health Care Campus, Haifa 3109601, Israel; m_plonsky@rmc.gov.il (M.P.-T.); d_magen@rmc.gov.il (D.M.); 2 Faculty of Medicine, Technion-Israeli Institute of Technology, Haifa 3109601, Israel

**Keywords:** chronic kidney disease, innate immune system, chronic inflammation, cardiovascular disease

## Abstract

Chronic kidney disease (CKD) constitutes a worldwide epidemic, affecting approximately 10% of the global population, and imposes significant medical, psychological, and financial burdens on society. Individuals with CKD often face elevated morbidity and mortality rates, mainly due to premature cardiovascular events. Chronic inflammation has been shown to play a significant role in the progression of CKD, as well as in the acceleration of CKD-related complications, including atherosclerosis, cardiovascular disease (CVD), protein–energy wasting, and the aging process. Over the past two decades, a substantial body of evidence has emerged, identifying chronic inflammation as a central element of the uremic phenotype. Chronic inflammation has been shown to play a significant role in the progression of CKD, as well as in the acceleration of CKD-related complications in dialysis patients, including atherosclerosis, CVD, protein–energy wasting, and the aging process. Remarkably, chronic inflammation also impacts patients with CKD who have not yet required renal replacement therapy. While extensive research has been conducted on the involvement of both the adaptive and innate immune systems in the pathogenesis of CKD-related complications, this wealth of data has not yet yielded well-established, effective treatments to counteract this ongoing pathological process. In the following review, we will examine the established components of the innate immune system known to be activated in CKD and provide an overview of the current therapeutic approaches designed to mitigate CKD-related chronic inflammation.

## 1. Contributing Factors to CKD-Related Immune System Activation 

The immune system’s activation in CKD has been attributed to multiple factors, with a large body of evidence demonstrating elevated blood levels of pro-inflammatory cytokines and chemokines and the activation of membrane and cellular components of innate immunity. Numerous factors are implicated in CKD-related immune activation, primarily including the following:

1. Gut dysbiosis: Alterations in gut bacterial composition due to restricted diet, chronic use of medications including potassium and phosphorus chelators, proton pump inhibitors, and recurrent bowel wall edema secondary to volume overload all contribute to metabolite changes that escalate inflammation, exacerbate oxidative stress, and promote renal fibrosis [1,2];
2. Lipid metabolism: The dysregulation of lipid metabolism has been observed in both animal models and CKD patients [3]. CKD is associated with a reduction in free fatty acid oxidation, increased lipid synthesis, and enhanced lipid gut uptake. Increased triglyceride synthesis augments the production of reactive oxygen species, thereby activating apoptotic and pro-fibrinogenic pathways [4];
3. Hypertension: Persistent high blood pressure is both a cause and a consequence of CKD. Adaptive and innate immunity have been both shown to participate in the pathogenesis of hypertension and ensuing kidney damage [5];
4. Water homeostasis disruption: Altered water homeostasis secondary to recurrent hypervolemia results in left ventricular heart remodeling, ventricular wall hypertrophy, ventricular length enlargement, and increased secretion of the brain natriuretic peptide (BNP) [6,7];
5. Waste product accumulation: The accumulation of waste products, including sodium, acids, phosphorus, urea, and medium-size molecules, alters the extracellular milieu, triggering immune activation [7];
6. Sympathetic overactivation: Elevated sympathetic activity may initiate and modulate inflammation in CKD through multiple pathways, including interferon γ, IL-6, and IL-10 [8];
7. Hemodialysis-related factors: Chronic hemodialysis exposes patients to substantial loads of dialysate water, with significant inter-dialytic variation in degrees of chemical purification microbiological quality and endotoxin levels, as well as in the biocompatibility of dialyzer membranes, potentially inducing an inflammatory state [9];
8. Inadequate medium-sized molecule removal: Conventional dialysis treatment is not sufficiently effective in the adequate removal of medium-sized molecules, the accumulation of which may trigger immune activation [10];
9. Immunomodulatory drug therapy: Medications targeting innate immunity cascade checkpoints, including NFκB and NLRP3 inflammasome, among others, as well as microbiota manipulation trials, which might give rise to a new era that is effective in diminishing chronic inflammation in CKD patients, with an effect on slowing the GFR decline curve.

## 2. CKD-Induced Activation of Innate Immunity

The interactions between renal parenchymal cells and resident immune cells, mediated by endothelial cell activation, result in the recruitment of circulating immune cells. This process leads to an inflammatory state, driven by the stimulation of Toll-like receptors (TLRs) and Nod-like receptors (NLRs) on resident immune cells. These receptors erroneously stimulate the danger-associated pathway of innate immunity through NFκB and NLRP3 inflammasome. Consequently, this cascade leads to the secretion of inflammatory mediators, causing tissue damage and progressive loss of function.

Studies have demonstrated enhanced endothelial activation in CKD as well as in CVD. This endothelial activation is characterized by increased levels of circulating cell adhesion molecules, including soluble Intracellular Adhesion Molecule 1 (sICAM-1), Vascular Cell Adhesion Molecule 1 (sVCAM-1), and sE-selectin [11]. Additionally, other endothelium-derived proteins such as monocyte chemoattractant protein-1 (MCP-1), angiopoietin-2, the tissue receptor (TR), and both a total and an active von Willebrand factor (vWF) have been observed to be increased. This state of endothelial activation is now recognized as a pivotal factor contributing to various cardiovascular morbidities associated with CKD, including atherosclerosis, increased arterial stiffness, IL-8-derived vascular calcifications, left ventricular hypertrophy, altered cardiac function, and reduced microcirculation. All these factors increase the risk of CVD morbidity and mortality in CKD patients, exceeding the traditional CVD risk factors within this patient population [6]. 

The innate immune system serves as the first line of defense against exogenous invasion, using a non-specific set of receptors that propagate a cascade of intracellular processes leading to a pro-inflammatory cytokine spillage from these cells. Subsequently, these cytokines recruit additional inflammatory cells, thereby further amplifying the inflammatory response and eventually leading to renal fibrosis and acceleration of CKD. Of note, the inflammatory reaction can also be initiated by endogenous factors. 

Pattern recognition receptors (PRRs) function as a key component of the innate immune system, playing a pivotal role in recognizing evolutionarily conserved molecular structures. PRRs are expressed in various cell types, including dendritic cells (DC), natural killer cells (NK), neutrophils, macrophages, fibroblasts, epithelial cells (including renal epithelium), and cells of the adaptive immune system. PRRs sense both pathogen-associated molecular patterns (PAMP), such as viruses, bacteria, or fungal nucleic acid (RNA or DNA), as well as danger-associated molecular patterns (DAMP), released from damaged cells following tissue injury. Upon detection, PRRs initiate either a pro-inflammatory or an anti-inflammatory response [6,12,13,14].

PRRs are divided into several classes, including TLRs, the C-type lectin receptor (CLR), the Retinoic acid-inducible gene (RIG)-I-like receptor (RLR), Absent in Melanoma-2 (AIM2), and the nucleotide-binding oligomerization domain (NOD)-like receptor (NLR). PRRs can be located either on the cell membrane surface or within the intracellular compartment. In response to PAMP or DAMP stimuli, NLRs and AIM2 assemble into multimeric cytosolic protein complexes, termed inflammasomes. Among the various inflammasomes, the NLRP3 inflammasome is the most extensively studied [15].

Upon activation of PRRs, three distinct molecular pathways are initiated:

1. Phagocytosis of the recognized PAMPs or DAPMs;
2. Local inflammatory signaling;
3. Activation of the adaptive immune system through the maturation of antigen-presenting cells.

## 3. TLR Signaling Pathway

TLRs are the most studied among PRRs. In humans, there are 10 functional TLRs known as TLR- through TLR10. TLR1, 2, 4, 5, 6, and 10 reside on the outer cell membrane in the form of either homodimers or heterodimers and are responsible for recognizing plasma components of pathogenic microorganisms, including lipopolysaccharides, lipid A, lipoproteins, and flagellin. TLR3, 7, 8, and 9 are expressed within intracellular vesicles, such as lysosomes, and act as sensors for microbial DNA and RNA, as well as specific proteins and lipids. Importantly, these receptors are also involved in the detection of endogenous substances released into the bloodstream following cellular injury [16].

Renal epithelial cells, including podocytes and tubular epithelium, as well as endothelial cells, macrophages, and dendritic cells within the kidney, all express TLRs and PRRs on their cell surface. Various types of renal injury were found to be mediated by TLR4 [17,18,19], indicating that TLR4 accelerates the injury process caused by nephrotoxic drugs [17], endotoxin-induced damage [19], and ischemia-reperfusion injury [20]. Conversely, the absence of TLR4 has been associated with significantly reduced cellular injury in response to these insults.

TLR signaling is activated by either the myeloid differentiation primary response gene 88 (MyD88) or the TIR-domain-containing adapter, including IFNβ (TRIF). Most TLRs (excluding TLR3) use the MyD88 signal as an adaptor molecule, promoting the activation of the nuclear factor kappa light chain enhancer (NF-κB) as well as the mitogen-activated protein kinase (MAPKs). Interleukin-6 (IL-6) and the tumor necrosis factor (TNF) are part of the NF-κB secretome [17].

Upon activation, MyD88-dependent TLRs initiate the assembling of the MyD88 and the IL1 receptor-associated kinase (IRAK) 1,2,4, which then leads to activation of the tumor necrosis factor receptor-associated factor 6 (TARF6), the activation of transforming growth factor beta activated kinase 1 (TAK1), and ultimately the activation of NF-κB and MAPKs, which induce inflammatory interleukins production. Employment of the TRIF pathway by TLR3 (or internalized TLR4) results in the activation of NF-κB and the interferon regulatory transcription factor 3 (IRF3), leading to the expression of type I IFN and pro-inflammatory cytokines such as pro-IL-1β and pro-IL-18 [12,21].

Activation of this pathway may occur due to several triggers, including TNF-, IL-1, Ang II, and the stimulation of NADPH oxidase (NOX)-dependent production of reactive oxygen species (ROS). This leads to rapid phosphorylation, site-specific ubiquitination, and subsequent degradation of the inhibitor of NF-κB (I-κB) protein by the 26S proteasome. The resulting free NF-κB molecules translocate to the nucleus and regulate target gene expression [22].

PAMPs bind microorganisms and classically activate TLR3, TLR5, TLR7, TLR8, and TLR11, leading to pathogen proteolytic lysis, while DAMPs activate TLR1, TLR2, TLR4, and TLR6 and lead to the macrophage removal of DAMPs. DAMP sterile inflammation causes the release of IL-1 cytokine, activating T cells that enhance natural killer cell (NK) cytotoxicity and macrophage phagocytosis function. They function not only in pathogen recognition but also in the recruitment of pathogen-eradication processes. The complement cascade and C-reactive protein (CRP) are soluble PRRs with the capacity for opsonization through the activation of the classical immune pathway [12].

The activation of NF-κB and activator protein 1 (AP-1) by TLR2 requires the formation of a heterodimer of TRL1 and TLR6. Upon activation, the TLR4 receptor assembles into a homodimer, while TLR2 is paired with a scavenger receptor such as CD36 as a co-receptor [14].

In their studies, Zewinger et al. [5] have shown that patients with CKD produce abnormal HDL, which activates endothelial TLR2 even in the absence of TLR1 and TLR6, which are normally needed for TLR2 activation. This activation led to increased ROS production via the SAPK/JNK-dependent activation of NOXs and not through the NF-κB pathway. The increased ROS production inhibited endothelial nitric oxide synthase (eNOS), thereby reducing endothelial NO bioavailability.

## 4. NLRP3 Pathway

Nucleotide-binding and oligomerization domain-like receptors (NLRs) are pro-inflammatory receptors of the PRRs that are located in the cytoplasm and have the ability to form multi-protein complexes known as inflammasomes, owing to their highly conserved NACHT domain. This assembly is triggered upon specific ligand recognition. NLRs, such as NOD, the leucine-rich repeat receptor kinase (LRR), and pyrin domain-containing protein 3 (NLRP3) also contribute to inflammasome formation through the cleavage of pro-caspase-1 to its active caspase-1 form. The inflammasome itself comprises a sensor receptor, NLRP3, an adaptor protein named the apoptosis-associated speck-like protein (ASC) containing a caspase recruitment domain (CARD), and procaspase-1. The activation of NLRP3 leads to oligomerization and the recruitment of ASC and procaspase-1, resulting in the formation of a multi-protein inflammasome complex, which self-activates caspase-1. Once activated, caspase-1 cleaves pro-IL-1β, pro-IL-18, and IL-33 into their mature forms, leading to the induction of pyroptosis, which represents an immune-related form of apoptosis in response to cellular infection by external pathogens and is characterized by cell swelling, hyper permeabilization of the plasma membrane, and ultimately rapid cell lysis [12,23]. Furthermore, activated caspase-1 plays a pivotal role in enhancing phagocytosis and bacterial killing by altering the pH of the phagosome via the NADPH oxidase NOX2 pathway [24].

The NLRP3 inflammasome has been implicated in the phenomenon of inflammaging, a process characterized by accelerated immunosenescence induced by chronic inflammation. This chronic inflammatory state arises from the sustained activation of innate immunity, commonly observed in CKD, acute kidney injury (AKI), diabetic kidney disease (DKD) [25], and crystal-related nephropathy. NLRP3 activation occurs in response to various endogenous signals, including uric acid, damaged mitochondria, aggregated proteins and lipids, and reactive oxygen species (ROS) [26,27]. These triggers for NLRP3 activation bear a striking resemblance to those activating TLRs, including the production of ROS and the thioredoxin-interacting protein.

**Figure 1:**
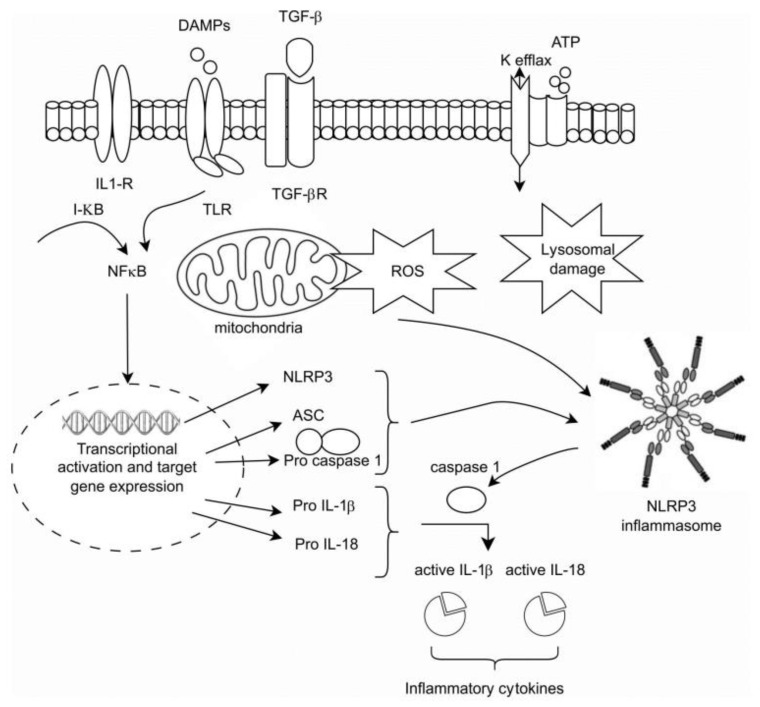
The intracellular activation of the innate pathway.

The activation of the NLRP3 inflammasome is dependent on a two-signal process: Signal 1 begins with the activation of membrane TLRs, as well as of PAMs, ROS, adenosine triphosphate, and other metabolites, collectively referred to as DAMPs, leading to the cytosolic activation of NF-κB, which, in turn, initiates nuclear transcription and the generation of pro-IL-1β and pro-IL-18. Signal 2 involves the assembly of NLRP3 and caspase-1 into an inflammasome, culminating in the cleavage of pro-IL-1β and pro-IL-18 by caspase-1 into their mature, biologically active forms [15] (Figure 1).

Experimental animal models of obstructive uropathy utilizing NLRP3-deficient mice have demonstrated diminished levels of inflammation attributed to reduced caspase-1 activation, along with the decreased renal maturation of IL-1β and IL-18. Furthermore, experiments involving bone marrow chimeras revealed that NLRP3 promotes renal injury and inflammation across both hematopoietic and nonhematopoietic cellular compartments [28]. Moreover, human renal biopsies from a wide variety of fibrotic kidney diseases showed increased mRNA expression of NLRP3. In addition, Mulay et al. have demonstrated that NLRP3-induced inflammation and fibrosis in calcium oxalate (CaOx) stone injury were ameliorated in the absence of NLRP3, caspase-1, and IL-1β [29].

NLRP3 exhibits functions extending beyond its inflammasome-related roles. In their experiments using CD4 T cells, Bruchard et al. [30] found NLRP3 to be located in the nucleus of T helper type 2 cells, in contrast to its previously reported cytosolic localization. Interestingly, NLRP3 was found to directly bind DNA within the promoter region of genes, functioning as a transcription factor in CD4 T cells, specifically activating T helper type 2 transcriptional programs. Chung et al. [31] reported the formation of a non-canonical NLRP3/ASC/caspase-8 complex at the mitochondria in response to tumor necrosis factor-α/cycloheximide stimulation downstream of tumor necrosis factor receptor signaling, contributing to renal tubular epithelial cell apoptosis. Furthermore, Anders et al. [32] demonstrated that NLRP3 augmented TGF-β–Smad signaling in renal fibroblasts in an inflammasome-independent manner, consequently promoting renal fibrosis. NLRP3 is expressed in a variety of kidney cell types, including podocytes, mesangial, and intercalated cells. Notably, IL-18 has been detected in the urine of patients experiencing AKI during episodes of acute tubular necrosis [22,33]. Moreover, there is evidence of elevated NLRP3 mRNA expression in kidney biopsies obtained from individuals with various fibrotic kidney diseases [12].

## 5. The NF-κB Role

In their animal model of hypertension, Henke et al. [22] reported that inhibition of NF-κB, specifically within endothelial cells, resulted in the declined progression of end-organ damage in CKD-related CVD, attributed to reduced local inflammation. This effect subsequently led to diminished damage in renal tubules, blood vessels, and, to a lesser extent, glomeruli, all without a significant alteration in arterial blood pressure. These findings are supported by the study of Muller et al. [34], which utilized the antioxidant pyrrolidine dithiocarbamate (PDTC) to inhibit NF-kB and found this inhibition to ameliorate inflammatory markers across glomerular, tubular, and collecting duct cells, as well as decrease levels of inflammatory infiltrates in renal and cardiac biopsies.

In the context of renal allografts, ischemia-reperfusion injury often triggers an inflammatory response that can sometimes culminate in rejection episodes. In a study by Vos et al. [35], it was demonstrated that the pre-treatment of donor kidneys with a competitive binding decoy for NF-κB led to a reduction in NF-κB activity and a subsequent decrease in macrophage cell infiltration within the tubulointerstitium. Furthermore, additional reports have demonstrated the benefits of NF-κB inhibition after infarction, owing to reduced cardiac remodeling and decreased infiltration of inflammatory cells in cardiac, renal, and vascular tissues [22,36,37].

Studies focused on hypertension have revealed the activation of shown inflammatory pathways. Bomfim et al. [38] observed that the anti-TLR4 antibody treatment of resistant arteries from spontaneously hypertensive rats led to a decrease in serum levels of TLR4, COX-2, and IL-6, suggesting an association between TLR4 and hypertension-related low-grade inflammation. Additionally, Dalekos et al. [39] have shown that circulatory levels of the NLRP3-dependent cytokine IL-1β are increased in patients with hypertension. In support of this, Droffel et al. [40] conducted experiments demonstrating that monocytes, when stimulated with Ang II, exhibit significantly higher secretion of IL-1β in peripheral blood monocytes from hypertensive patients compared to healthy individuals. Notably, the angiotensin 1 (AT1) receptor blocker, valsartan, was found to reduce IL-1β secretion by monocytes from hypertensive patients [41]. 

Pang et al. [42] have reported that Irisin, a recently identified myokine secreted from skeletal muscle during exercise, plays a crucial role as an inhibitor of pyroptosis and cell death in smooth muscle cells of the aorta in mice with CKD.

### Can the Local Renal Inflammatory Process Be Contained for Improved Patient Outcomes?

**Figure 2:**
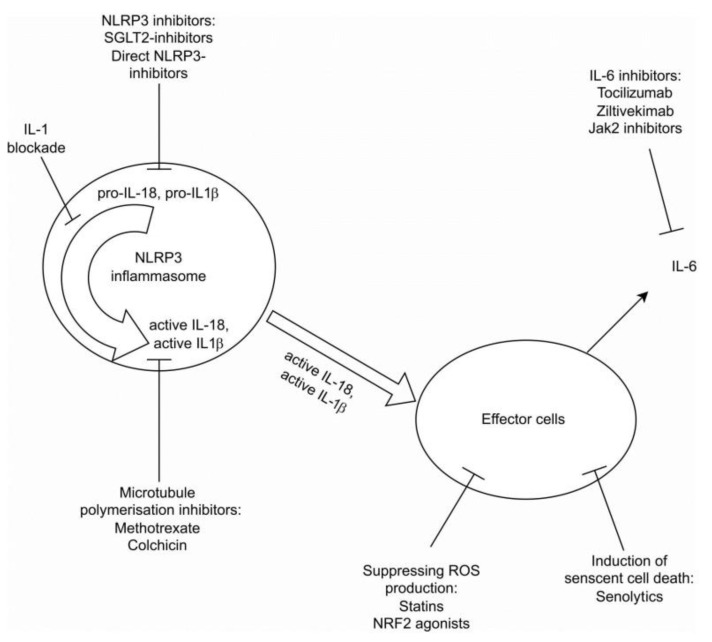
Summary of current therapeutic approaces.

Numerous anti-inflammatory medications have been evaluated for their potential to reduce inflammation, thereby mitigating both CVD events and the progression of CKD [3,5,14,43]. In the following sections, we will examine several of these drugs and review the conducted studies related to them:

1. Statins: Statins have a well-established role in reducing inflammation in patients with CKD and its associated CVD [3,5,43]. Treatment with atorvastatin has been shown to reduce inflammatory markers in CKD patients, independent of its cholesterol-lowering effects, suggesting its anti-inflammatory/antioxidant properties [44]. Combining other lipid-lowering drugs, such as bempedoic acid and ezetimibe, may offer additional therapeutic benefits;
2. Sodium Glucose Cotransporter 2 (SGLT2) Inhibitors: SGLT2 inhibitors are known for their nephroprotective and cardiovascular benefits in patients with CKD [3,14,45]. Emerging evidence suggests they may also harbor an anti-inflammatory role, with indications of their capacity to inhibit the NLRP3 inflammasome and reduce inflammatory markers [46];
3. Finerenone and Glucagon-like Peptide (GLP) 1 Agonists: Finerenone, a mineralocorticoid receptor antagonist, has demonstrated nephroprotective effects and reduced cardiovascular events in the FIDELIO DKD trial. Both finerenone and GLP-1 agonists have been shown to exert anti-inflammatory effects [14,47,48,49];
4. Anti-IL-1R: The CANTOS trial investigated the use of canakinumab in inhibiting inflammation mediated by NLRP3-IL1-IL6 pathways. It showed a reduction in cardiovascular event rates and slowed disease progression in both CKD and non-CKD patients. The drug effect was directly related to the magnitude of downstream reduction in IL-6 and CRP levels [5,14,45,50]. However, its positive impact on renal function preservation was modest [3,14,42,48,49];
5. IL-6 Inhibition: Inhibition of IL-6, a downstream effect of IL-1β, has been proposed to block the inflammatory response [3,14]. Tocilizumab is an IL6R inhibitor that was shown to reduce troponin and CRP in the setting of NON-ST elevation myocardial infarction. Zilitivekimab, a fully humanized anti-IL6 monoclonal antibody developed specifically for the prevention of atherosclerotic damage in CKD patients, was shown in the RESCURE trial [51] to reduce CRP levels in these patients. The currently ongoing ZEUS study (Ziltivekimab the drug impact on cardiovascular outcome study) aims to explore the drug’s effect on both cardiovascular events and kidney function [52];
6. Colchicine and Low-Dose Methotrexate: These non-specific anti-inflammatory agents have broad effects related to microtubule polymerization, reducing leukocyte chemotaxis, cytokine secretion, and inhibiting NLRP3 pathways [14,43]. Colchicine has been shown to reduce cardiovascular events in the COLCOT [53] and LoDoCo2 trials [54], though gastrointestinal side effects were reported. In a meta-analysis [55], colchicine treatment was associated with a lower risk of major cardiovascular events, though a mild trend was noted towards higher non-cardiovascular mortality for unknown causes. Unlike the beneficial effect of colchicine, low-dose methotrexate did not show a cardiovascular benefit in a NIH-funded trial [56];
7. NRF2 Agonists: Nrf2 is an anti-oxidative and anti-inflammatory transcription factor that is reduced in CKD and considered a potential therapeutic target [3,14]. Several Nrf2 agonists have been developed and studied. Older studies demonstrated the possible benefit of these agents; for example, butylhydroquinone significantly improved the phosphate-induced calcification of vascular smooth muscle cells via the activation of KEAP1/NRF2/P62 signaling and repressing ROS production [57]. Dimethyl fumarate also had similar vascular outcomes in vitro and in vivo [58]. Another Nrf2 agonist, hydrogen sulfide, showed similar in vitro results [59]. Bardoxolone methyl was studied in patients with CKD and diabetes mellitus type 2 [60,61], showing conflicting results and necessitating premature trial cessation. Additional Nfr2 agonists are under evaluation [14];
8. Senolytics: Cellular senescence is associated with inflammation and tissue damage. Senolytics, which induce the death of senescent cells, hold potential anti-inflammatory properties [14]. This hypothesis has been studied through various kidney injury experimental models, including ischemia-reperfusion injury, diabetic nephropathy, and mouse kidney transplantation models, indicating improved cell repair and reduced proteinuria and fibrosis. In the phase one trial examining the effect of the tyrosine kinase inhibitor dasatinib, combined with the plant flavanol quercetin in diabetic nephropathy, results showed reduced cell senesce and diminished levels of the pro-inflammatory cytokines IL-1a and IL-6. Moreover, ABT-63, a potent B cell lymphoma (Bcl)2/w/Xl inhibitor, effectively depleted senescent cells when administered orally to mice and awaits further studies [62]. Another ongoing Mayo Clinic study, currently in process, examines the effect of fisetin, a dietary supplement with a presumably antioxidant effect [63], on the reduction in inflammatory markers, mesenchymal cell changes, and the GFR decline rate in patients with CKD and DM2. The use of senolytics for diverse medical indications is under investigation, showing potential as promising future therapies [64]. Figure 2 summarizes the above-mentioned therapeutic approaches.

Another area of interest in targeting the immune system is dietary intervention and modulation of the microbiome. So far, no single intervention has shown to significantly and consistently reduce inflammatory response in patients with CKD, but evidence from small randomized controlled trials did show that probiotics, prebiotics, phosphate binder sevelamer, dietary fibers, and antibiotics may lower the concentration of uremic toxins and reduce inflammatory markers, making it an interesting future subject of research [14,43].

It is important to note that while these treatments hold promise, their effectiveness and safety may vary in different patient populations and stages of kidney disease. Further research and clinical trials are necessary to establish their roles in managing inflammation and improving outcomes in CKD and related conditions.

Furthermore, it is worth emphasizing that adopting a healthy lifestyle, including regular exercise, smoking cessation, maintaining a balanced diet, and achieving a healthy body weight, is a well-known and beneficial intervention for cardiovascular diseases and kidney-related damage. These lifestyle choices have the potential to contribute to the reduction in inflammatory biomarkers [3].

## 6. Future Developments

Future anti-inflammatory approaches are being developed and studied in parallel with the ever-growing knowledge regarding the innate immune system and its signaling pathways.

1. Anti-NRP3 treatments, either genetic-based or pharmacological, are being studied, both in CKD and CVD, rheumatological, gastrointestinal, and neurological conditions, and more [3,14,43,65];
2. JAK2 inhibitors have also gained popularity in targeting the immune system in several diseases, mainly inflammatory, rheumatological, and hematological. It has an important role in the IL6 signaling pathway [3,14];
3. Other various approaches can be of potential benefit and are being evaluated, including modulation of immune checkpoints, inhibition of NET formation, modulation of chemokines signaling, leukocyte–endothelial adhesion, and macrophage activity [3,5,14,66].

## 7. Targeting Fibrosis

Fibrosis is a hallmark of CKD and represents an independent factor in disease progression, regardless of the primary insult. Whether fibrosis is mainly a cause or a consequence of CKD progression remains a subject of debate. Accumulated data from animal models may not be applicable to humans [67]. Aside from the uncertainty regarding the pathogenic role of kidney fibrosis, there are no optimal methods to assess kidney fibrosis in clinical practice, and only a limited number of clinical trials assess kidney fibrosis as their primary endpoint [66,67]. These caveats underscore the challenges and gaps in our understanding of fibrosis in the context of kidney disease and the need for further research to develop better diagnostic tools and therapeutic strategies for managing renal fibrosis.

In the pathogenicity of fibrosis, the inflammatory process, initiated by the innate immune system in response to injury, exacerbates tissue damage through the release of cytokines and free radicals, further leading to parenchymal microvascular dysfunction and aberrant wound-healing response [68]. Innate immune signaling in fibroblasts and their precursors is directly linked to fibrogenesis. In addition, genetic variations and resulting cellular cross-talk play vital roles in the fibrosis process. Targeting the innate immune system in order to reduce fibrogenesis is intriguing. While TGFβ has been considered the canonical ligand driving fibrogenesis through the body, recent studies have raised doubts regarding this view. Rather, metabolic and immunologic responses are emerging as major activators of the process that should be targeted [67,68].

Despite not being classic immune cells typically derived from circulating leukocytes, fibroblasts produce a wide array of pro-inflammatory cytokines, chemokines, and oxygen radicals. In diseased tissue, they release large amounts of these substances and are now presumed to be key regulators of the immune system response. It has been shown that fibroblasts are more sensitive to DAMPs (from injured tissue) than macrophages, resulting in higher levels of a pro-inflammatory milieu. Genetic studies have pointed towards a predisposition for fibrotic changes in response to injury with specific polymorphisms through the dysregulation of innate immunity. Examples include a polymorphic change in IL1β and IL1 receptor-associated kinase (IRAK1) loci and coding polymorphisms in TLR4, which are associated with CKD. Therefore, it is suggested that inhibiting the innate immune system’s signaling may help prevent fibrosis and advanced renal damage [3,67].

Several trials have shown successful anti-fibrotic effects with the blockade of the innate immune system [3,67,68]. For example, the blockade of FN14 signaling within the TNF superfamily receptors was effective. Similarly, inhibition of TLRs and downstream molecules such as IRAK1 and 4 slowed fibrosis progression after injury. The JAK/STAT pathway, recently linked to the risk of fibrosis progression in diabetic nephropathy, is also a target for blockade in early trials [68].

As mentioned above, clinical trials have not been able to demonstrate the efficacy of the TGFβ blockade in halting fibrosis progression. Thus, there is evidence of activation of multiple pathways that can drive fibroblast persistence, including WNT, Notch, Hedgehog, and innate immune signaling, including IL6, IL8, IL1β, and interferon, as well as pro-inflammatory chemokines. These pathways may be intriguing targets for inhibition [68]. Baricitinib, a JAK1/2 inhibitor, has demonstrated a reduction in proteinuria in diabetic nephropathy during a 6-month trial and may be a promising agent for future studies [3,68].

Recent FDA approvals of two novel anti-fibrotic drugs, pirfenidone and nintedanib, for the treatment of idiopathic pulmonary fibrosis suggest potential benefits in slowing kidney fibrosis progression [3,67,68]. Pirfenidone has anti-inflammatory and anti-fibrotic effects, primarily regulating TNF pathways and modulating cellular oxidation, resulting in the downregulation of growth factors and procollagens 1 and 2. Ongoing clinical trials are exploring its roles in FSGS, diabetic nephropathy, and various diseases underlying CKD [3,67,68].

Nintedanib is a multikinase inhibitor that potently blocks both PDGRα and PDGRFβ. Blocking these receptors appears to be beneficial in fibrotic diseases, though previous experience with the kinase inhibitor imatinib provided conflicting results [3,67,68]. In the kidneys, both PDGRα and PDGRFβ receptors are expressed by fibroblasts, making nintedanib a promising candidate for halting progressive kidney disease. Accumulating data from in vitro trials and animal models of autosomal dominant polycystic kidney disease [69] and obstructive uropathy [70] have shown beneficial effects of nintedanib as an anti-fibrotic agent. However, its side effects, including reported cases of nintedanib-induced glomerular microangiopathy, may limit its use [71].

Additional novel therapeutic approaches include antagomirs (lademirsen) or drugs targeting IL11 or NKD2 (a WNT singling pathway inhibitor). Another potential therapeutic approach involves targeting tubular dysfunction, which contributes to fibrosis. During the search for novel therapies, some drug developments have been halted due to side effects, financial reasons, or other factors. Examples of drugs that were developed for the prevention of kidney fibrosis but were withdrawn from further development include Fresolimumab, FG-3019, STX-100, and GCS-100 [3,67,68].

## 8. Summary

There is a growing understanding of the innate immunity cascade and its role in the ongoing damage observed in CKD. The innate immune system, through PRRs, senses cell damage and triggers a cascade of intracellular protein activation. This process leads to local inflammation and recruits the adaptive immune system, collectively accelerating inflammation. Ultimately, this chronic inflammation eventually contributes to fibrosis and the deterioration of renal function.

Treatments that target specific molecules within this cascade have shown partial efficacy, and some have caused severe side effects, leading to the termination of certain clinical trials. This may be attributed to the conservative role of the innate immune system.

Through understanding the main pathways involved in the inflammatory process of CKD, several questions arise: What makes CKD different from most other chronic diseases that do not activate the immune system in this manner? How can we effectively control inflammation to prevent organ fibrosis? Is there a molecule that can effectively “turn off” the devastating inflammatory process in the kidneys without interfering significantly with the essential functions of the inflammatory system?

Gaining more insights into the immune system’s response to other chronic diseases may shed light on potential interventions for CKD and guide the development of therapies that preserve an appropriate balance between suppressing inflammation and preserving its vital roles.

## Data Availability

No new data were created.
